# Performance of five molecular methods for monitoring *Arcobacter* spp

**DOI:** 10.1186/1471-2180-13-220

**Published:** 2013-10-03

**Authors:** Arturo Levican, María José Figueras

**Affiliations:** 1 Unitat de Microbiologia, Departament de Ciències Mediques Bàsiques, Facultat de Medicina i Ciències de la Salut. IISPV, Universitat Rovira i Virgili, Reus, Spain

**Keywords:** *Arcobacter*, Identification, Comparison, Molecular methods, 16S rRNA-RFLP, m-PCR, 23S rRNA, *gyrA*

## Abstract

**Background:**

Bacteria belonging to the *Arcobacter* genus are emerging enteropathogens and potential zoonotic agents. Their taxonomy has evolved very rapidly, and there are presently 18 recorded species. The prevalence of species belonging to *Arcobacter* is underestimated because of the limitations of currently available methods for species identification.

The aim of this study was to compare the performance of five PCR based methods that target regions of 16S rRNA, 23S rRNA or *gyrA* genes to identify *Arcobacter* species, and to review previous results reported in the literature using these methods.

**Results:**

The five tested methods were found not to be reliable. They misidentified between 16.8% and 67.4% of the studied strains; this was dependent upon the target regions of the tested genes. The worst results obtained were for the identification of *Arcobacter cryaerophilus* and *Arcobacter butzleri* when the 23S rRNA gene was used as the target. These species were confused with many non-targeted species.

**Conclusion:**

Our results suggest that the known diversity of *Arcobacter* spp. in different environments could be expanded if reliable identification methods are applied in future studies.

## Background

*Arcobacter* spp. are emerging enteropathogens and potential zoonotic agents that can be transmitted by food and water [1]. Previous studies have demonstrated a relationship between the presence of arcobacters in water samples and bacterial indicators of faecal pollution [2,3]. This genus belongs to the *Campylobacteraceae* family and was originally proposed by Vandamme *et al*. in 1991 [4] to accommodate two aerotolerant species (*Arcobacter cryaerophilus* and *Arcobacter nitrofigilis*), which had previously been included in the *Campylobacter* genus. Since 2009, the number of newly described species has risen exponentially, and the genus currently comprises 18 species, eight of which were described in our laboratory [1,5-8].

The identification of *Arcobacter* spp. by phenotypic testing is difficult. This is because they can easily be confused with *Campylobacter* spp. [1,9]. This has led to the design of many different molecular detection and identification methods. These are based on conventional PCR, multiplex PCR (m-PCR), Real Time PCR (RT-PCR), Restriction Fragment Length Polymorphism (RFLP), Denaturing Gradient Gel Electrophoresis PCR (DGGE-PCR), Fluorescence in situ Hybridisation (FISH) and Matrix Assisted Laser Desorption Ionization Mass Spectrometry (MALDITOF MS); these methods are reviewed by Collado & Figueras [1]. The majority of PCR based methods [10-13] target the genus, or only *Arcobacter butzleri* and/or *A. cryaerophilus*[1] and references therein]. Others also include *Arcobacter skirrowii*[14,15] or *Arcobacter cibarius*[16]. In 2010, Douidah *et al*. proposed a new m-PCR method that could identify the five species associated with humans or other mammals, i.e. *A. butzleri, A. cryaerophilus, A. skirrowii, A. cibarius* and *Arcobacter thereius*[9]*.* This m-PCR method was not able to detect *Arcobacter trophiarum*, which was originally isolated from pigs by De Smet *et al*. [17]. Therefore, the authors proposed a PCR method targeting the *hsp60* gene for this species. In 2008, Figueras *et al*. [18] designed an RFLP identification method based on the digestion of the 16S rRNA gene with the *Mse*I endonuclease; this was able to identify the six species so far described (*A. butzleri, A. cryaerophilus, A. cibarius, A. skirrowii, A. nitrofigilis*, and *Arcobacter halophilus*). This method was recently updated with the inclusion of additional endonucleases (*Mnl*I and *Bfa*I), and is able to identify the 17 *Arcobacter* spp. described at the time of publication [19]. The prevalence of *Arcobacter* spp. in different matrices such as water, food, and faeces is underestimated because of the limitations of the identification methods used to recognize all species [1]. Despite this, no study has comparatively evaluated the performance of the most commonly used identification methods. The aim of this study was to test the performance of five molecular identification methods across all *Arcobacter* spp. The compared methods were selected because they target a higher number of *Arcobacter* species [9,14-18]. Furthermore, a literature review was performed to analyse the results that have been obtained using these methods since their publication.

## Methods

The five identification methods were compared using 95 different strains, these included type and reference strains, as well as field strains. These strains represented all currently accepted *Arcobacter* species (Additional file 1: Table S1), but did not include the recently described *Arcobacter anaerophilus*[8]. The five molecular methods investigated were selected because they targeted a higher number of species. They were as follows: two m-PCRs designed for *A. butzleri, A. cryaerophilus*, and *A. skirrowii*[14,15]; a PCR method that targets *A. butzleri, A. cryaerophilus, A. skirrowii*, and *A. cibarius*[16]; and two methods that target *A. butzleri, A. cryaerophilus, A. skirrowii*, *A. cibarius*, and *A. thereius* (the m-PCR method described by Douidah *et al*. [9]), or *A. nitrofigilis* and *A. halophilus* (the 16S rRNA-RFLP method described by Figueras *et al.*[18])*.* As the *A. trophiarum* PCR identification of De Smet *et al*. [17] was designed to complement the previously published m-PCR of Douidah *et al*. [9], both methods were considered to be a single one when evaluating their performance (Tables 1 and 2).

**Table 1:** **Performance of five molecular methods used for the identification of *Arcobacter *species in relation to a reference method**^**a**^

		**Houf *et al*. [**[14]**]**			**Kabeya *et al*. [**[15]**]**			**Figueras *et al*. [**[18]**]**			**Pentimalli *et al*. [**[16]**]**			**Douidah *et al. *[**[9]**]**		
														**De Smet *et al*. [**[17]**]**^**b**^		
**Targeted species**	**Strains**^**c**^	**A**	**B**	**C**	**A**	**B**	**C**	**A**	**B**	**C**	**A**	**B**	**C**	**A**	**B**	**C**
***A. butzleri***	21	16S	100	0	23S	4.8	6	16S	100	3	16S	100	4	23S	100	4
***A. cryaerophilus***	19	23S	100	11	23S	100^d^	8	16S	63.2	0	*gyrA*	100	1	*gyrA*	100	1
***A. skirrowii***	5	16S	100	4	23S	100	3	16S	100	0	*gyrA*	60	2	23S	100	0
***A. cibarius***	8							16S	100	0	*gyrA*	0^e^	0	23S	100	0
***A. thereius***	5													23S	100	0
***A. trophiarum***	3													*hsp60*	100	0
***A. nitrofigilis***	5							16S	100	0						
***A. halophilus***	1							16S	100	0						

(A) targeted genes, (B) percentage of correctly identified strains of the targeted species, and (C) number of non-targeted species misidentified as targeted ones.

^a^All strains were identified using the RFLP method of Figueras *et al*. [19] specifically designed to recognize all species.

^b^The method designed by De Smet *et al.*[17] only detects or identifies *A. trophiarum*, and was intended to complement the m-PCR of Douidah *et al.*[9]. Therefore, they are grouped together as a single method.

^c^The strains of the nine *Arcobacter* species not listed in this table (n=28) belong to new species that were not targeted by the compared methods.

^d^The method was designed to differentiate subgroups 1A and 1B of this species, but not all strains of these subgroups were well recognized (Table 2).

^e^Despite the eight strains of *A. cibarius* being correctly assigned to this species, none of them was considered to be correctly identified. This is because they were all confused with *A. butzleri*, and three of them with *A. skirrowii*, when using primers that targeted those species (Table 2).

**Table 2:** **Identification results obtained for 95 strains of 17 *Arcobacter *spp. when using the five different PCR identification methods**

**Species**	**Strains**^**a**^	**Houf *et al*. [**[14]**]**	**Kabeya *et al*. [**[15]**]**	**Figueras *et al.* [**[18]**]**^**b**^	**Pentimalli *et al*. [**[16]**]**	**Douidah *et al.* [**[9]**]**
						**De Smet *et al*. [**[17]**]**^**c**^
***A. butzleri *(Ab)**	21	21 Ab	1 Ab^d^	21 Ab	21 Ab	21 Ab
			15 Ab + Acr1B^e^			
			5 NA^f^			
***A. cryaerophilus *(Acr)**	19	19 Acr	19 Acr	12 Acr	19 Acr	19 Acr
				7 Ab		
**Acr1A (n=6)**			5 Acr1A^d^	6 Acry1A^d^		
			1 Acr1B			
**Acr1B (n=6)**			5 Acr1B	6 Acry1B		
			1 Acr1A			
***A. skirrowii *(Aski)**	5	5 Aski	5 Aski	5 Aski	3 Aski^d,g^	5 Aski
					2 NA	
***A. nitrofigilis *(Anit)**	5	5 Aski	4 Acr1B^d^	5 Anit	2 Ab	NA
			1 Ab + Acr1B		2 Acr	
					3 NA*^d^	
***A. halophilus *(Ahalo)**	1	1 Aski + Acr	1 Aski	1 Ahalo	NA*	NA
***A. cibarius *(Acib)**	8	8 NA	3 Aski^d^	8 Acib	8 Ab	8 Acib
			5 Aski + Acr1B		8 Acib	
					3 Aski	
***A. thereius *(Ather)**	5	5 Acr	1 Ab	5 Ab	5 NA*	5 Ather
			2 Ab + Acr1B^d^			
			1 Acr1B			
			1 NA			
***A. mytili *(Amyt)**	3	3 Aski	3 Aski	3 Amyt	3 NA*	3 NA
***A. marinus *(Amar)**	1	1 Acr	1 NA	1 Amar^h^	1 Ab	1 NA
***A. molluscorum *(Amoll)**	3	3 Aski + Acr	3 NA	3 Amoll	3 NA*	3 NA
***A. defluvii *(Adef)**	11	11 Acr	11 Ab	11 Adef	11 NA*^d^	11 Ab
***A. trophiarum *(Atroph)**	3	3 Acr	2 Ab^d^	3 Ab	3 NA*	3 Atroph
			1 NA			
***A. ellisii *(Aelli)**	3	3 Acr	3 Acr1A + Acr1B	3 Aelli	2 Aski	1 Ab
					1 NA*^d^	2 Ab +Acr^d^
***A. bivalviorum *(Abiv)**	3	3 Acr	3 Acr1B	3 Abiv	3 NA	3 NA
***A. venerupis *(Aven)**	1	1 Acr	1 Ab	1 Aven^h^	1 Ab	1 Ab
***A. cloacae *(Acloa)**	2	2 Acr	2 Ab + Acr1B	2 Acloa	2 NA*	2 NA
***A. suis *(Asuis)**	1	1 Acr	1 Acr1A	1 Adef	1 NA	1 Ab
**Correctly identified strains**		53 (55.8%)	31 (32.6%)	79 (83.2%)	79 (83.2%)	79 (83.2%)

^a^All strains were identified using the RFLP method of Figueras *et al*. [19] that had been specifically designed to recognize all species. The new species *Arcobacter anaerophilus* was not tested as it had only recently been described [8]. However, a computer simulation of the digestion of the 16S rRNA gene sequence of the type strain of this species (Accession number FR686494) using the *Mse*I endonuclease [18,19] showed a species-specific RFLP pattern (658, 138, 60, 52, 41, 34, 28, 12, 3 bp).

^b^As this method was designed for *A. butzleri, A. cryaerophilus, A. cibarius, A. skirrowii, A. nitrofigilis* and *A. halophilus*[18]*,* the results for strains of other species were interpreted based on the RFLP patterns described in subsequent publications [5-7,23-25].

^c^The method designed by De Smet *et al.*[17] only detects or identifies *A. trophiarum*, and was intended to complement the m-PCR of Douidah *et al.*[9]. Therefore, they are grouped together as a single method.

^d^Result obtained for the type strain.

^e^Species A + species B refers to the fact that the expected amplicon for species A and B were obtained in the same reaction.

^f^NA or NA*: No amplification of a band of the expected size, or (*) band/s of another size were obtained.

^g^When different results were obtained using the four individual PCR reactions designed by Pentimalli *et al*. [16] for *A. butzleri, A. cryaerophilus, A. skirrowii*, and *A. cibarius,* they are shown on separate lines.

^h^*A. venerupis* produced a pattern very similar to that of *A. marinus*[19].

All tested strains were grown on 5% sheep blood agar for 48 h at 30°C under aerobic conditions. DNA was extracted using the InstaGene DNA Purification Matrix (Bio-Rad Laboratories, Hercules, CA, USA), and quantified using GeneQuant (Amersham Pharmacia Biotech, Cambridge, England) following the manufacturer’s instructions. PCR amplifications were carried out in a 2720 Thermal Cycler (Applied Biosystems, Carlsbad, CA, USA) using the primers and conditions described in the different studies (Additional file 1: Table S2). The identity of all field strains was confirmed in a previous study using the 16S rRNA-RFLP method described by Figueras *et al*. [19].

The evaluation of the performance of the methods was based on the percentage of strains of the targeted species that were correctly identified, and on the number of non-targeted species that gave erroneous results (Tables 1, 2 and Additional file 1: Table S1).

The literature review was carried out following PRISMA guidelines [20], using the Citations Search tool in the Web of Science® V 5.8 in the Thomson Reuters ISI Web of Knowledge research platform (http://www.accesowok.fecyt.es). The platform was accessed using the Spanish national license via the *Fundación Española para la Ciencia y la Tecnología* (FECYT), and was last accessed on July 30th 2012. Each of the five studied molecular methods was searched by author, topic (*Arcobacter*), and year of publication to obtain the total number of citations for each method since publication until 2012. Citations were analyzed individually to find the total number of strains identified at the species level. The number of strains of each species identified using any of the compared methods, were used to make the calculations shown in Additional file 1: Table S3. In studies where no genotyping method was used, it was assumed that each isolate represented a strain.

## Results and discussion

### Comparative performance of the five molecular methods

The percentage of correctly identified strains obtained using the five identification methods, and the number of misidentified non-targeted species greatly depended upon the method used (Tables 1 and 2). The percentage of misidentified strains ranged from 16.8% to 67.4% (Table 2). The m-PCR method of Kabeya *et al*. [15] had the worst performance, and produced unreliable results for all three of its targeted species (Tables 1 and 2). Although all strains of *A. cryaerophilus* and *A. skirrowii* were correctly identified, a further eight and six non-targeted species, respectively, were mistakenly identified as one of these two species (Table 1). Furthermore, only 4.8% of the *A. butzleri* strains were correctly identified, with six non-targeted species being confused with this species (Tables 1 and 2). Globally, the Kabeya *et al.* m-PCR method correctly identified just 32.6% (31/95) of the studied strains. Although this method was also designed to differentiate subgroups 1A and 1B of *A. cryaerophilus*, not all strains of these subgroups were correctly identified (Table 2). This correlates with the *in silico* observations of Douidah *et al*. [9] who reported that the primer used [15] were not specific enough to provide correct identification of *A. cryaerophilus* at the subgroup level. Further to this, Debruyne *et al.*[21] have suggested, that based on results of AFLP and *hsp60* analyses, the subgroup nomenclatures 1A and 1B should be abandoned.

The second least reliable method analysed was the m-PCR technique described by Houf *et al.*[14]. This correctly identified 55.8% (53/95) of the strains (Table 2), including all those belonging to its targeted species (*A. butzleri, A. cryaerophilus*, and *A. skirrowii*; Table 1). This method was 100% reliable for the identification of *A. butzleri*, and there was no confusion with other species. However, nine of the fourteen non-targeted species generated the typical amplicon of *A. cryaerophilus*; two that of *A. skirrowii*; and two simultaneously generated both amplicons (Tables 1 and 2)*.* Only *A. cibarius* produced no amplification when using this method (Table 2). These results agree with previous studies that showed the existence of misidentifications when using this method [1,5-7].

A similar number of correctly identified strains (83.2%) were obtained when using the other three evaluated methods (Pentimalli *et al.*[16]; the combined method of Douidah *et al*. [9] and De Smet *et al.*[17]; and Figueras *et al.*[18]). However, the number of misidentified non-targeted species differed depending upon the method used (Tables 1 and 2). Most misidentification occurred when using the method of Pentimalli *et al.*[16]. In this case, four non-targeted species were confused with *A. butzleri,* one with *A. cryaerophilus*, and two with *A. skirrowii* (Tables 1 and 2). Furthermore, the expected amplicons for *A. butzleri* and *A. skirrowii* in individual reactions were also obtained for the eight and three strains of *A. cibarius*, respectively (Table 2). Nevertheless, no cross-reaction with non-targeted species occurred when using primers designed for *A. cibarius* that reacted only with the eight strains of this species. The combined method of Douidah *et al.*[9] and De Smet *et al.*[17], misidentified four of the non-targeted species (*Arcobacter defluvii, Arcobacter ellisii, Arcobacter venerupis*, and *Arcobacter suis*) as *A. butzleri*, and two of the three strains of *A. ellisii* as *A. cryaerophilus* (Table 2). The method performed correctly for the four remaining targeted species. Finally, the 16S rRNA-RFLP designed by Figueras *et al*. [18] was found to misidentify three species (*A. trophiarum*, *A. thereius*, and some *A. cryaerophilus* strains) as *A. butzleri*. Further to this, *A. suis*, and *A. defluvii* produced the same pattern, and two species (*A. venerupis*, and *Arcobacter marinus*) a very similar one (Table 2). Because of these limitations, this method has recently been updated with new endonucleases; these produced specific results for all strains and species [19]. This updated protocol was the one used to identify all strains used in this study.

### Comparative evaluation of the targeted genes and designed primers

When the results were evaluated in relation to genes used to identify the species, it was observed that the 23S rRNA gene regions targeted in the Kabeya *et al.*[15] method for *A. butzleri, A. cryaerophilus*, and *A. skirrowii* were unreliable, as was the region employed in the Houf *et al*. method [14] for *A. cryaerophilus* (Tables 1 and Additional file 1: Table S2). However, the regions of the 23S rRNA gene targeted by the m-PCR method of Douidah *et al*. [9] were 100% reliable for the detection of *A. skirrowii, A. cibarius*, and *A. thereius*, but not for *A. butzleri* (Tables 1, 2 and Additional file 1: Table S2). With regard to the *gyrA* gene, the region used for the identification of *A. cryaerophilus* in the latter method, and the one in the method of Pentimalli *et al*. [16] were unreliable. Despite all strains of *A. cryaerophilus* being correctly identified, *A. ellisii* was confused with this species when using the Douidah *et al.*[9] method, and with *A. skirrowii* when using the Pentimalli *et al*. [16] method (Tables 1 and 2). The main reason for the poor performance of the targeted regions of 23S rRNA or *gyrA* genes (Additional file 1: Table S2) is the limited amount of sequences used to derive the primers. For instance, the sequences of the 23S rRNA gene are, at the time of writing, only available for eight of the seventeen known *Arcobacter* species (*A. butzleri, A. cryaerophilus, A. skirrowii, A. cibarius, A. nitrofigilis, A. thereius, Arcobacter mytili*, and *A. trophiarum*), and the *gyrA* gene is only available for seven species (*A. butzleri, A. cryaerophilus, A. skirrowii, A. cibarius, A. nitrofigilis, A. marinus*, and *A. halophilus*). In contrast, the sequences of the 16S rRNA gene are available for all species of the genus, and this has enabled the identification of endonucleases that produce specific patterns for all species; as described in the recently published update of the 16S rRNA-RFLP method [19]. The 16S rRNA gene has also been used to design specific primers for *A. skirrowii* and *A. butzleri* in the Houf *et al*. method [14], and for *A. butzleri* by Pentimalli *et al*. [16]. However, only the primers that targeted the 16S rRNA region chosen by Houf *et al*. [14] for the identification of *A. butzleri* (Additional file 1: Table S2) were 100% specific*,* and showed no cross-reaction with other species (Tables 1 and 2).

### Literature review of the studied methods

The results of the literature review, which summarised the total number of strains and species identified using any of the five compared methods (Additional file 1: Table S3), revealed that the m-PCR method of Houf *et al*. [14] was the most globally referenced, with 71.9% (123/171) of all citations. This method was used to identify 64.8% (2735/4223) of the strains recorded in the literature since 2000 (Additional file 1: Table S3). The next most frequently used methods were the 16S rDNA-RFLP of Figueras *et al*. [18] and the m-PCR of Douidah *et al.*[9], which were used to identify 14.6% and 13.4% of strains, respectively (Additional file 1: Table S3). The overall most prevalent species were *A. butzleri* (63.7% of strains), followed by *A. cryaerophilus* (27.3%), and *A. skirrowii* (4.9%) (Additional file 1: Table S3). The other 14 species represented only 4.1% of the recovered strains (Additional file 1: Table S3). The species diversity may be influenced by the different origins of the strains and/or the isolation methods used in the analysed studies.

When considering the results obtained in the present study, with those of the literature review, the strains identified as *A. butzleri* (64.5%) using the m-PCR designed by Houf *et al*. [14] could be considered to be correctly identified (Additional file 1: Table S3). However, the use of this method has probably led to a global overestimation of the number of *A. cryaerophilus* and *A. skirrowii* as some of the strains identified are likely to belong to other species (Tables 1 and 2). For example, when Atabay *et al.*[22] used the Houf *et al*. method [14] they identified six *A. skirrowii* strains that were not able to hydrolyze indoxyl acetate, despite this being a typical phenotypic characteristic of the species. Interestingly, *A. mytili,* one of only two *Arcobacter* species (along with *Arcobacter molluscorum*) unable to hydrolyze indoxyl acetate, produces the typical band of *A. skirrowii* when the m-PCR method of Houf *et al*. [14] is used. Therefore, the six strains identified by Atabay *et al.*[22] may belong to that species. Further evidence for this confusion of results can be found in a study on the prevalence of *Arcobacter* in meat and shellfish in which strains belonging to *A. nitrofigilis* and *A. thereius* were recognized [23]. This is because contradictory results were seen when using two identification methods in parallel [14,18]. When using the Houf method [14], *A. nitrofigilis* produced the expected amplicon for *A. skirrowii* and *A. thereius* the amplicon expected for *A. cryaerophilus*. However, when using the method of Figueras *et al*. [18] the expected 16S rRNA-RFLP pattern of *A. nitrofigilis* and *A. butzleri* was obtained for the *A. nitrofigilis and A. thereius* strains, respectively. The correct identity of these strains was confirmed as *A. nitrofigilis* and *A. thereius* through sequencing of the 16S rRNA and/or *rpoB* genes [23]. This sequencing approach resolved the discrepancies observed between the two identification methods [14,18] and has also led to the discovery of the species *A. mytili, A. molluscorum, A. defluvii, A. ellisii, Arcobacter bivalviorum, A. venerupis, A. cloacae,* and *A. suis*[5-7,24-26].

The use of the m-PCR method of Douidah *et al.*[9] in combination with the PCR method of De Smet *et al.*[17] enabled *A. thereius* (17.6%, 100/567), *A. trophiarum* (1.8%, 10/567), and *A. cibarius* (0.2%, 1/567) to be recognized in two independent studies [27,28] (Additional file 1: Table S3). Nevertheless, there is a weakness in this approach as the strains of four non-targeted species may be misidentified as the more frequently isolated *A. butzleri* (Tables 1 and 2).

Finally, with regard to studies that used the methodology designed by Kabeya *et al*. [15], our results revealed that all of the targeted species may have been overestimated; this is because 12 of the 14 non-targeted species could be misidentified (Tables 1 and 2). No studies were found that used the PCR method of Pentimalli *et al*. [16], and our results indicate that this method is not reliable (Tables 1 and 2).

## Conclusion

In this study, the performance of five different PCR methods used to identify all known *Arcobacter* spp. has been compared for the first time. None of the compared methods were completely reliable, and they displayed different misidentification rates for both targeted and non-targeted species; many of which have been described after the publication of the method. The current study has highlighted the limitations of the compared methods. We consider the way forward to be the use of the more reliable methods in parallel for verification of identity of the isolates. Our results suggest that the currently known diversity of *Arcobacter* spp. in different environments will change in the future as reliable identification methods, such as the updated 16S rRNA-RFLP method [19], are applied.

## Competing interests

The authors declared that they have no competing interests.

## Authors’ contributions

AL carried out the experiments, the literature review, and was the principal author of the manuscript. MJF designed the research project, evaluated results, helped draft the manuscript, and supervised AL. Both authors read and approved the final manuscript.

## Supplementary Material

Additional file 1: Table S1 Strains of *Arcobacter* spp. used in the study. **Table S2.** Targeted genes and PCR conditions of the compared methods. **Table S3.** Literature review of 171 studies (2000–2012) that identified 4223 strains of *Arcobacter* using the five compared PCR methods. Click here for file
