# Supplementary material for: Performance of five molecular methods for monitoring Arcobacter spp
Source: BMC Microbiol. 2013 Oct 3;13:220. doi: 10.1186/1471-2180-13-220 (PMC3850767; doi:10.1186/1471-2180-13-220)
Supplement: Additional file 1: Table S1 — Strains of Arcobacter spp. used in the study. Table S2. Targeted genes and PCR conditions of the compared methods. Table S3. Literature review of 171 studies (2000–2012) that identified 4223 strains of Arcobacter using the five compared PCR methods. [file 1471-2180-13-220-S1.pdf]

Table S1 Strains of *Arcobacter* spp. used in the study.

SPECIES	STRAIN	SOURCE
A. bivalviorum **n=3**	F4[T,a,b], F118-2[a,b], F118-4[a,b]	Mussels
A. butzleri **n=21**	LMG 10828[T,a,b], LMG 11118[b]	Human faeces
	F42, F46[a,b], F49, F51	Pork
	F15, F22, F23, F24, F25	Turkey
	F47, F52	Chicken
	F50[b], F53	Beef
	F1, F2, F29, F30, F98-1	Mussels
	T62	Soil
A. cibarius **n=8**	CECT 7203[T,a,b]	Chicken
	NC81[b], NC88[b]	Piggery effluent
	H742, H743[b], H745, H746[b], H748	Poultry carcasses
A. cloacae **n=2**	SW28-13[T,a,b]	Sewage
	F26[a,b]	Mussels
A. cryaerophilus **n=19**	LMG 9904[T,a,b], LMG 9871[a]	Bovine abortion foetus
	LMG 9865[a,b], LMG 10241[b], LMG 6622, LMG 10229[a,b]	Porcine abortion
	LMG 9065[a], LMG 7537[a], LMG 9863[a,b]	Ovine abortion foetus
	LMG 10829[a]	Human blood
	LMG 9861[a,b]	Bovine abortion foetus
	FE4[a,b], FE5[a,b], FE6[a,b], FE9[a,b], FE11[a], FE13[a], FE17[a]	Chicken faeces
	FE14[b],	Ovine faeces
A. defluvii **n=11**	CECT 7697[T,a,b], SW28-7[a,b], SW28-8, SW28-9, SW28-10, SW30-2[a,b], SW30-7, SW30-8	Sewage
	CC42[b]	Pig faeces
	CH8-2, SAN599-9[b]	Mussels
A. ellisii **n=3**	F79-6[T,a,b], F79-2[a,b], F79-7[a,b]	Mussels
A. halophilus **n=1**	LA31B[T,a,b]	Hypersaline lagoon
**A. marinus** **n=1**	CECT 7727[T,a,b]	Seawater/starfish

A. molluscorum		
**n=3**	CECT 7696[T,a,b], F91[a,b], F101-1[a,b]	Mussels
A. mytili		
**n=3**	CECT 7386[T,a,b], CECT 7385[a,b]	Mussels
	T234[b]	Brackish water
A. nitrofigilis		Roots of *Spartina*
**n=5**	CECT 7204[T,a,b], LMG 7547[b]	*alterniflora*
	F39[b], F40[¶], F72[b]	Mussels
A. skirrowii		
**n=5**	LMG 6621[T,a,b]	Lamb faeces
	LMG 9911	Porcine abortion
	Houf 989[a,b], Houf 994[b]	Cow faeces
	S7[b]	Sludge
A. suis		
**n=1**	F41[T,a,b]	Pork
A. thereius		Porcine abortion
**n=5**	LMG 24486[T,a,b], LMG 24487[a,b]	foetus
	SW24[b]	Sewage
	F61-1[b]	Pork
	F93-4[b]	Clams
A. trophiarum		
**n=3**	LMG 25534[T,a,b], LMG 25535[a,b]	Pig faeces
	CECT 7650[a,b]	Chicken cloaca
A. venerupis		
**n=1**	F67-11[T,a,b]	Clams

ATCC: American Type Culture Collection. LMG: Belgian Co-ordinated Collection of Microorganisms. CECT: Colección Española de Cultivos Tipo.
[a]Strains from which the 16S rRNA gene was sequenced for verification of identity [19].
[b]Strains from which the *rpoB* gene was sequenced for verification of identity [19].

Table S2 Targeted genes and PCR conditions of the compared methods.

Author	Targeted species (expected amplicon)	Targeted gene: Position (nt)[a]	Primers (pmol)	Concentrations[b,c]	Conditions
Houf *et al*. [14]	*A. butzleri* (400)<br>*A. cryaerophilus* (230)<br>*A. skirrowii* (640)	16S rRNA: 959 – 1357<br>23S rRNA: 1720 – 1964<br>16S rRNA: 705 - 1357	ARCO (50)<br>BUTZ (50)<br>CRY1 (50)<br>CRY2 (50)<br>SKIR (25)	$MgCl_2$ 1.3 mmol l^{-1}<br>*Taq* DNA polymerase 1.5 U	Initial denaturation 94°C, 2 min and final extension 72°C, 5 min<br>32 Cycles of: Denaturation 94°C, 45 s; Annealing 61°C, 45 s; Chain extension 72°C, 30 s
Kabeya *et al*. [15]	*A. butzleri* (692)<br>*A. cryaerophilus* 1A (728)<br>*A. cryaerophilus* 1B (152)<br>*A. skirrowii* (448)	23S rRNA: 1174 - 1865<br>23S rRNA: 1135 – 1865<br>23S rRNA: 1713 – 1865<br>23S rRNA: 1423 - 1865	N.c.1A (25)<br>ARCO-U (25)<br>N.butz (2.5)<br>N.c.1B (2.5)<br>N.ski (2.5)	MgCl2 1.5 mmol l-1<br>Taq DNA polymerase 2.5 U	Initial denaturation 94°C, 3 min and final extension 72°C, 5 min<br>30 Cycles of: Denaturation 94°C, 30 s; Annealing 62°C, 60 s; Chain extension 72°C, 60 s
Figueras *et al*. [18]	Species specific patterns for species[d]	16S rRNA: 47 - 1073	CAH 1am (25)<br>CAH 1b (25)	MgCl2 1.5 mmol l-1<br>Taq DNA polymerase 2.5 U	Initial denaturation 94°C, 2 min, final extension 72°C, 10 min<br>30 Cycles of: Denaturation 94°C, 30 s; Annealing 52°C, 30 s; Chain extension 72°C, 90 s
Pentimalli *et al*. [16]	*A. butzleri* (203)<br>*A. cryaerophilus* (212)<br>*A. skirrowii* (257)<br>*A. cibarius* (145)	16S rRNA: 803 – 1006<br>*gyrA*: 2337 – 2549<br>*gyrA*: 1366 – 1622<br>*gyrA*: 2364 - 2778	16S Arcobutz (30)<br>Gyr Arcocry (30)<br>Gyr Arcoski (30)<br>Gyr Arcocib (50)<br>All F and R	MgCl2 2.0 mmol l-1<br>Taq DNA polymerase 2.0 U	Initial denaturation 94°C, 2 min and final extension 72°C, 7 min<br>40 Cycles of: Denaturation 94°C, 60 s; Annealing 55°C, 60 s; Chain extension 72°C, 60 s
Douidah *et al*. [9]	*A. butzleri* (2061)<br>*A. cryaerophilus* (395)<br>*A. skirrowii* (198)<br>*A. cibarius* (1125)<br>*A. thereius* (1590)	23S rRNA: 646 - 2707<br>*gyrA*: 2255 – 2640<br>23S rRNA: 646 – 844<br>23S rRNA: 646 - 1771<br>23S rRNA: 646 - 2236	ButR (50)<br>SkiR (50)<br>TheR (50)<br>CibR (50)<br>ArcoF (50)<br>GyrasF (50)<br>GyrasR (50)	MgCl2 1.5 mmol l-1<br>Taq DNA polymerase 1.5 U	Initial denaturation 94°C, 2 min and final extension 72°C, 10 min<br>30 Cycles of: Denaturation 94°C, 45 s; Annealing 58°C, 45 s; Chain extension 72°C, 2 min
De Smet *et al*. [17]	*A. trophiarum* (382)	*hsp60*: 686 - 1068	hsp60F and R (50)	MgCl2 1.4 mmol l-1<br>Taq DNA polymerase 2.0 U	94°C, 3 min, final extension 72°C, 5 min<br>30 Cycles of: Denaturation 94°C, 45 s; Annealing 58°C, 45 s; Chain extension 72°C, 30 s
Figueras *et al*. [19]	Species specific patterns for the different species[d]	16S rRNA: 47 - 1073	CAH 1am (25)<br>CAH 1b (25)	MgCl2 1.5 mmol l-1<br>Taq DNA polymerase 2.5 U	Initial denaturation 94°C, 2 min, final extension 72°C, 10 min<br>30 Cycles of: Denaturation 94°C, 30 s; Annealing 52°C, 30 s; Chain extension 72°C, 90 s

[a] Positions of 16S rRNA gene are based on *Escherichia coli*, and those of the 23S rRNA and *gyrA* genes on sequences present in the genome of *A. butzleri* RM4018.

[b]All PCRs included dATP, dCTP, dGTP, and dTTP (Applied Biosystems(TM)) at a concentration of 200 µM each, 5 µl 10X Invitrogen(TM) buffer, and Milli-Q water in a final volume of 50 µl. The Taq DNA polymerase was supplied by Invitrogen(TM).

[c]For the m-PCR of Kabeya *et al*. (15) 50 µg of DNA was used, and 100 µg for the other methods.

[d]Digestion of the 16S rRNA obtained amplicon (1026 bp) with the different endonucleases *Mse*I [18], *Mse*I, *Mnl*I, or *Bfa*I [19] generated species specific RFLP patterns for the 17 tested species of the genus.

Table S3 Literature review of 171 studies (2000-2012)[a] that identified 4223 strains of *Arcobacter* using the five compared PCR methods.

	**Number of Identified strains (%) of the different species in relation with the method used**				
**Species**	**No (%)**	**Houf *et al*. [14]**	**Kabeya *et al*. [15]**	**Figueras *et al*. [18]**	**Douidah et al. [9]<br>De Smet et al. [17][b]**
A. butzleri	2690 (63.7%)	1763 (64.5%)	241 (79%)	445 (72.2%)	241 (42.5%)
A. cryaerophilus	1152 (27.3%)	850 (31.1%)	45 (14.8%)	112 (18.2%	145 (25.6%)
A. skirrowii	209 (4.9%)	87 (3.2%)	19 (6.2%)	33 (5.4%)	70 (12.3%)
**Other *Arcobacter* spp.**	172 (4.1%)	35 (1.3%)[c]	---	26 (4.2%)[c]	111 (19.6%)[d]
**Total**	4223	2735 (64.8%)	305 (7.2%)	616 (14.6%)	567 (13.4%)

[a]Of the 171 references found using the ISI Web of Knowledge (last access on July 30th 2012), Houf *et al.* [14] was cited in 123 studies; Kabeya *et al*. [15] in 21; Douidah *et al*. [9]/De Smet *et al*. [17] in 11; and Figueras *et al*. [18] in 16. Pentimalli *et al*. [16] was cited three times, but used only for background information.
[b]The method designed by De Smet *et al.* [17] only detects or identifies *A. trophiarum*, and was intended to complement the m-PCR of Douidah *et al.* [9]. Therefore, they are grouped together as a single method.
[c]The remaining 14 *Arcobacter* spp. were detected using other molecular identification methods such as 16S rRNA or *rpoB* gene sequencing and/or 16S rRNA-RFLP [17,18] used in parallel [5,6,7,23,24,25,26].
[d]These strains included 100 *A. thereius*, 10 *A. trophiarum*, and 1 *A. cibarius* (0.2%, 1/567) that were identified by De Smet *et al*. [27,28] using this combination of methods.
